# Forward step down test - clinical rating is correlated with joint angles of the pelvis and hip: an observational study

**DOI:** 10.1186/s12891-023-06943-4

**Published:** 2023-10-12

**Authors:** Smadar Peleg, Ruth Pelleg-Kallevag, Yuval Almog, Gideon Herman, Oren Nakdimon, Michal Arnon, Gali Dar

**Affiliations:** 1 Levinsky-Wingate Academic College (Wingate Campus), Netanya, Israel; 2 Department of Physical Therapy, Zefat Academic College, Zefat, Israel; 3 Faculty of Medicine and Health, University of Sydney, Camperdown/Darlington, NSW Australia; 4 Physical Therapy Clinic, Maccabi Healthcare Services, Modi’in, Israel; 5 Department of Physical Therapy, Faculty of Social Welfare and Health Sciences, University of Haifa, Haifa, Israel; 6 Physical Therapy Clinic, The Ribstein Center for Sport Medicine Sciences and Research, Wingate Institute, Netanya, Israel

**Keywords:** Clinical evaluation, Functional tests, Forward Step Down Test, Single leg squat, Visual observation

## Abstract

**Background:**

Clinical methods for assessing quality of movement and functional tests are important to clinicians. Typical deviations from normal kinematics during the clinical test of Forward Step Down Test (FSDT) are pelvic tilt and hip adduction which are associated with the risk of knee pain.

**Objectives:**

(1) to examine the correlation between clinical assessment of the FSDT and joint angle measurements of pelvis, hip, knee and ankle joints in males and females; (2) to examine the differences in joint angles between individuals rated as good, fair or poor in a FSDT performance test.

**Methods:**

Ninety-two healthy individuals performing FSDT were video-taped with two-dimensional digital video cameras. The clinical assessment of the FSDT was rated by two experienced physical therapists as good, fair, or poor based on a Crossley et al. (2011) validated scale. Measurements of pelvic drop, hip adduction and knee valgus were taken using Image J software.

**Results:**

Out of 177 lower limbs, 74 (37 in each limb) were clinically rated as “good/fair” (41.80%) while 103 (52 in the dominant leg and 51 in the non-dominant leg) were rated as “poor” (58.19%). No significant differences were observed between dominant and non-dominant legs or between males and females in clinical rating of the FSDT. Pelvic drop angle was significantly higher and hip adduction angle was significantly lower for “poor” clinical rating compared to “good/fair” in both dominant and non-dominant legs (p < 0.001) in males and females. Females demonstrated higher pelvic drop, lower hip adduction and higher knee valgus angles compared with males (p < 0.05).

**Conclusions:**

This study showed that the clinical rating of FSDT is correlated with joint angle measurements suggesting that this assessment can be utilized in clinical practice. Individuals with poor quality performance of FSDT showed higher pelvic drop and hip adduction movement. Further studies examining different populations with diverse disorders or pathologies are essential.

**Supplementary Information:**

The online version contains supplementary material available at 10.1186/s12891-023-06943-4.

## Background

Performance tests are frequently employed by physical therapists to clinically screen the individual’s status and functional ability and to monitor his/her progress during the rehabilitation process. The aim of performance tests is to simulate real-time activity executed by the individual and to assess his/her movement pattern [1].

The importance of performance/functional tests is linked to the notion that faulty movements during functional activities are related to a greater risk of injuries, for example: altered movement such as knee valgus during jumping or squatting increases the risk of anterior cruciate ligament injuries and of anterior knee pain or patellofemoral pain, while pelvic drop is greater among individuals with patellofemoral and hip pain [2–8]. In addition, some studies have found a correlation between muscle weakness and faulty movement such as, trunk deviation during walking when abductor muscles are weak or altered gait pattern [8–11].

Thus, early identification of altered or faulty movement during performance or functional tests might reduce the risk of injury or assist when establishing a rehabilitation program following an injury.

The Forward Step Down Test (FSDT), involves stepping down from a stair in order to enable the visual assessment of movement quality during weight bearing on one leg while performing flexion and extension of the knee [12, 13]. During the test, observation is being performed to evaluate joints alignment and neuromuscular control. The most common scale evaluating the FSDT was developed by Crossley and others in 2011 [14]. This clinical evaluation of FSDT performance includes: an overall impression as to the ability to maintain balance, trunk posture, pelvis position, hip joint position, and knee joint alignment [14]. The examiner rates the movement as “good”, “fair” or “poor”. The test was found to have good reliability [13, 14].

The advantage of the FSDT is the ability to perform an easy and direct visual observation which can be adapted to the field or the clinic without any special technology [15]. Yet, although it is used by clinicians, the FSDT is a subjective assessment and examining the association to objective measurements is essential.

It has been previously found that there are sex differences in kinematics at the pelvis, hip, and knee during different activities suggesting different movement strategies between males and females [15–17]. For example, Weeks et al. (2015), found that joint angles of pelvic rotation and hip adduction were smaller among men compared with females during single leg squat; Gracci et al. (2012) also found greater hip adduction and knee abduction among women, less trunk flexion and higher trunk rotation [16, 18]. Hence, it should be considered whether these discrepancies between males and females affect performance and test scores.

Therefore, our aims were: (1) to examine the correlation between clinical assessment of the FSDT and joint angle measurements of pelvis, hip, knee and ankle joints in males and females; (2) to examine the differences in joint angles between individuals rated as good, fair or poor in a FSDT performance test.

## Methods

### Study design

Clinical rating of FSDT performance and angular measurements of the pelvis and hip in the frontal plane of healthy young males and females were carried out based on video recording and Image J software (v.1.51) (Image Processing and Analysis in JAVA) [19].

### Setting

#### Study procedures

The subjects’ height, weight and BMI, as well as their leg dominance (determined as the leg used to kick a ball), were recorded. Following a short warm-up (cycling for five minutes on a stationary bike), the following anatomical landmarks were marked on each participant on both sides: the anterior superior iliac spine (ASIS), the mid patella, and the mid-line between the lateral and medial malleoli (representing the center of the ankle joint). All the subjects wore only underwear, thereby exposing all these anatomical landmarks. The subjects performing FSDT were videotaped with two-dimensional digital video cameras (JVC Everio GZ-HD5EK HD, Japan/USA). One camera was placed on a tripod, three meters in front of the subject, at a height of one meter and two other cameras were placed 2 m laterally to the participant’s legs (Supplementary 1) [13, 14, 20].

#### The forward step down test (FSDT)

**Fig. 1:**
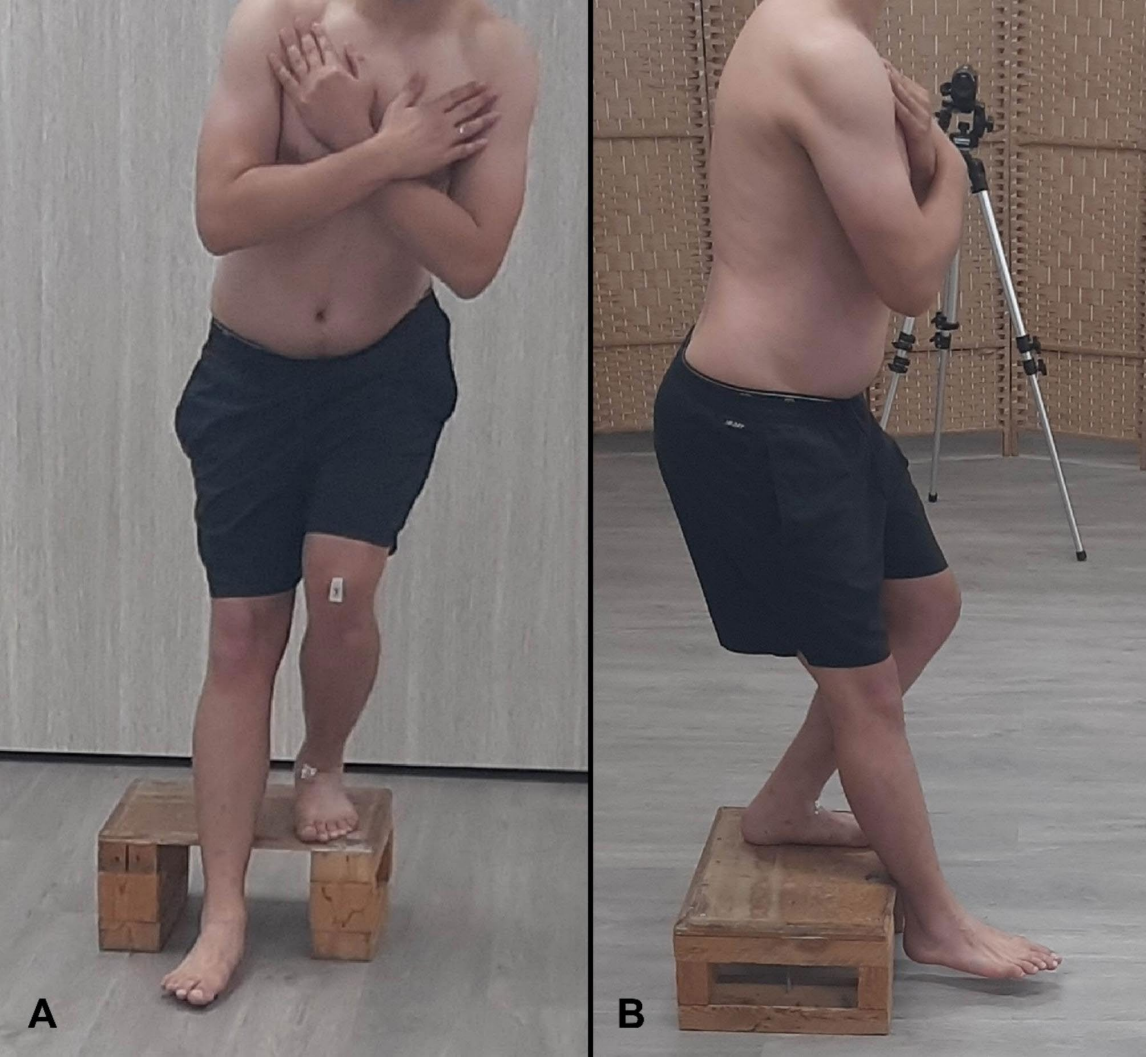
Forward Step Down Test in the frontal plane (**A**) and sagittal plane (**B**)

The subjects stood on a 20 cm high step with arms across their chest, and were instructed to step down to the floor while keeping their balance on the weight-bearing leg (Fig. 1). Once initial heel contact was made with the floor, the subject was instructed to return to the starting position, and perform five consecutive repetitions at a rate of one step-down per 3 s. Three practice trials were performed and after two minutes of rest, the FSDT was performed [13, 14].

#### Clinical rating of the FSDT

Two physical therapists (both with over 10 years of clinical experience) evaluated all video recordings and rated the quality of the FSDTs. Clinical rating was based following Crossley et al.’s (2013) scale: an overall impression as to the ability to maintain balance, trunk posture (i.e., trunk lateral deviation or shift, trunk rotation, trunk lateral flexion, trunk forward flexion), the position of the pelvis (i.e., pelvic lateral deviation, rotation, or pelvic tilt), hip joint (i.e., hip adduction, hip internal rotation), and knee joint (i.e., knee valgus) in space. The examiner graded the performance as good, fair, or poor [14].

### Bias

All videos were viewed independently by both examiners, who then compared their assessments. In the event of discrepancies, the two examiners re-evaluated the recording, discussed the differences, and reached a final decision. Before the data collection, both examiners received several hours of training during which they practiced and discussed the different segments of the scale and its implementation based on 10 examples of the FSDTs videotaped earlier.

#### Reliability of the FSDT rating and joint angles

Reliability tests for the clinical evaluation and the joint angle measurements were conducted prior to data collection. Intra-observer measurements were taken twice by the same researcher from 15 individuals, with a two-week interval between the sessions. Inter-observer measurements from 15 individuals were taken simultaneously by two independent researchers (YA and DS), blinded to each other’s results.

### Participants

A total of 92 healthy individuals (48 males and 44 females; mean age 25.7(± 2.9)) volunteered for this study. Subjects were included if they were pain-free and presented no musculoskeletal or neurological disorders affecting their lower extremities or lumbar spine during the six months preceding the study. Any subject suffering from dizziness secondary to the use of medication that could cause loss of balance, was excluded [13]. The study was approved by the Human Research Ethics Committee of Zefat Academic College, Israel (N. 04-2017). All participants signed an informed consent form. The participants were recruited by advertisement among Zefat Academic College physical therapy students, in Zefat, Israel.

### Variables

#### Joint angle measurements during the FSDT

**Fig. 2:**
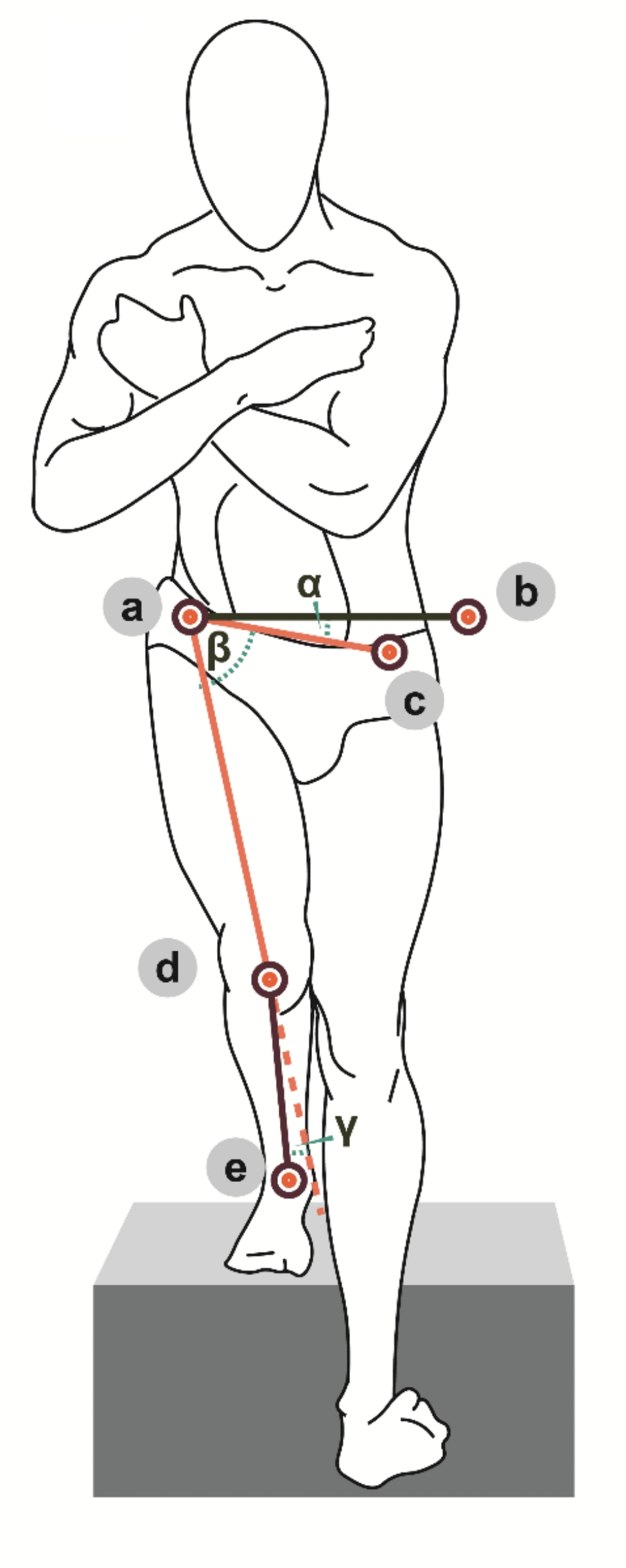
Pelvic drop (α angle), hip adduction (β angle) and knee valgus (γ angle) measurements

The following measurements were performed from the frontal plane: the pelvic tilt, hip adduction and knee valgus angles. The pelvic drop angle (α) was measured between a line connecting both anterior superior iliac spines (ASISs) and a horizontal line running from the ASIS [21, 22]. The hip adduction angle (β) was measured between a line connecting both ASISs and another line from the ASIS to the center of the patella [23]. A greater angle represents lower adduction movement. The knee valgus angle (γ) was measured as the angle created between a line running from the ASIS to the center of the patella and a line running between the center of the patella and the center of the ankle joint/mortise (between the two malleoli) (Fig. 2) [24].

### Data sources/measurements

All measurements were taken by the same two physical therapists using Image J software (v.1.51) (Image Processing and Analysis in JAVA) [19]. The measurements were taken while the heel of the forward leg reached the floor (implying maximal knee flexion of the standing leg) and were performed five times, during each of the FSDT repetitions. The average was recorded and saved for further analysis.

### Study size

Prior to data collection, the sample size was calculated using G*power 3.1 software. The alpha level was set at 0.05, and a power of 80%. In addition, considering the fact that FSDT performance was rated according to three grades, it was determined that 159 lower limbs were required for the study.

### Quantitative variables

For descriptive statistics, quantitative variables were described with mean (± SD). Weighted kappa coefficient (k) was used for intra- and inter-observer agreement of the FSDT clinical ratings and the intra-class correlation coefficient (ICC) (ICC model 2,1), analysis for the joint angle measurements. Weighted kappa values were interpreted as follows: <0.40 - poor agreement, 0.41–0.60 -moderate agreement, 0.61–0.80 - substantial agreement, and 0.81-1 – almost perfect [25]. ICC values were as follows: <0.40 - poor agreement, 0.40–0.59 – fair agreement, 0.60–0.74 – good agreement, 0.75-1 – excellent agreement [26].

Due to the small number in the “good” performance group, the “good” and “fair” performances were grouped as “fair/good” performance cohort and were compared to the “poor” performance cohort (Supplementary 2 – Data unanalyzed values).

### Statistical methods

Each dependent variable was examined for normality assumption via skewness (*SK* < [2.0]) and kurtosis (*K* < 7.00) procedures. Skewness values ranged between − 0.005 and 0.675 and kurtosis values ranged between − 0.028 and 0.583. Therefore, a normal distribution was assumed for dependent variables.

The chi square test compared the FSDT clinical ratings between males and females and between the dominant and non-dominant leg of the same individual.

For each leg (dominant and non-dominant), a multivariate analysis of variance (MANOVA) was performed on the variables within each cluster, including pelvic drop, hip adduction, knee valgus, sex and interaction between variables. This procedure was followed by an ANOVA. Tukey’s post hoc multiple comparison tests were performed when the F-test was significant (*p* < 0.05).

We performed binomial logistic regression to predict FSDT performance. In the regression model, we assessed the explanatory power of (a) pelvic drop, (b) hip adduction, (c) knee valgus on the accuracy of FSDT clinical rating for the dominant and the non-dominant leg.

The chi square test compared the FSDT clinical ratings between the dominant and non-dominant leg of the same individual in order to evaluate leg symmetry.

Data were analyzed using the SPSS (v. 26.0) program. Significance was set at *p* < 0.05.

## Results

Eighty-nine dominant legs (96.7%) and 88 non-dominant legs (95.7%) were analyzed due to poor visualization of at least one of the markers in 7 out of 184 cases.

### Demographic characteristics

Descriptive data of the studied population is summarized in Table 1. Out of 92 subjects, 75(81.5%) reported their right leg as dominant (40 males, 35 females). All parameters were significantly higher in males compared with females, beside age which was controlled (20–30) (Table 1).

**Table 1:** Demographic characteristics (mean (± SD)) of the studied population (t-test)

	Females (n = 44)	Males (n = 48)	Total (n = 92)	p-value
Age (year)	25.04 (± 8.70)	26.43 (± 2.79)	25.7(± 2.9)	0.557
Height (m)	162.01 (± 4.89)	175.01 (± 6.54)	169.2(± 8.4)	< 0.001*
Weight (kg)	57.65 (± 8.97)	75.33 (± 11.70)	66.88(± 13.66)	< 0.001*
BMI (kg/m2)	21.68 (± 3.08)	24.51 (± 3.02)	23.26(± 3.34)	< 0.001*

*Significant differences (P < 0.05)

### Reliability tests

The reliability of the clinical rating of FSDT was almost perfect, with intra-observer agreement of quadratic weighted kappa of 0.960 (95% confidence interval: 0.9126-1; p < 0.001) and inter-observer agreement of quadratic weighted kappa variation of 0.918 (95% confidence interval: 0.797-1, p < 0.001).

The reliability analysis of joint angles measurements demonstrated excellent agreement of the intra-observer variation, i.e., ICC = 0.938 (95% confidence interval: 0.870–0.971, p < 0.001) and inter-observer variation, i.e., ICC = 0.911 (95% confidence interval: 0.821–0.956, p < 0.001).

### Clinical ratings of the FSDT

Out of 177 lower limbs, 74 (37 in each limb) were clinically rated as “good/fair” (41.80%) and 103 (52 in the dominant leg and 51 in the non-dominant leg) were rated as “poor” (58.19%). No significant differences were observed in the clinical ratings (″good/ fair″ vs. ″poor″) when tested for sex effect, of the FSDT (Table 2). No differences between dominant and non-dominant legs were observed in the FSDT results (*p* = 0.310). Therefore, further analysis was performed on the entire sample (177 lower limbs in total).

**Table 2:** A comparison of clinical rating by sex (chi squared test, p < 0.05)

Males (N = 94)				Females (N = 83)				Total (N = 177)				P value between sexes for dominant leg		P value between sexes for non-dominant leg	
Dominant leg (N = 47) N (% within sex)		Non-Dominant leg (N = 47) N (% within sex)		Dominant leg (N = 42) N (% within sex)		Non-Dominant leg (N = 41) N (% within sex)		Dominant leg (N = 89) N (%)		Non-Dominant leg (N = 88) N (%)					
Fair and Good	Poor	Fair and Good	Poor	Fair and Good	Poor	Fair and Good	Poor	Fair and Good	Poor	Fair and Good	Poor				
20 (42.6%)	27 (57.4%)	22 (46.8%)	25 (53.2%)	17 (40.5%)	25 (59.5%)	15 (36.6%)	26 (63.4%)	37 (41.6%)	52 (58.4%)	37 (42.0%)	51 (58.0%)	0.507	0.226		

### Joint angle measurements and clinical rating

The mean joint angle measurements of pelvic drop, hip adduction and knee valgus angles for the dominant and non-dominant leg limbs of the entire sample were: 7.44° (± 3.71) and 6.69° (± 3.58); 75.11° (± 7.14) and 76.78° (± 8.10); 4.20° (± 2.53) and 4.06° (± 2.91), respectively.

Pelvic drop was significantly higher and hip adduction was significantly lower for “poor” clinical rating compared to “good/fair” in both dominant and non-dominant legs (p < 0.001) in males and females (Table 3). Significant differences were found between males and females implying higher pelvic drop, lower hip adduction and higher knee valgus angles among females (p < 0.05). No interaction was found between sex and clinical rating for any joint angle measurements (Table 3).

**Table 3:** A comparison of pelvic drop, hip adduction and knee valgus angles (Mean ± SD) according to FSDT clinical rating, sex and leg dominance (MANOVA, p < 0.05)

Variable		males (n = 48)				females (n = 44)				p value		
		Clinical rating Good/fair		Clinical rating Poor		Clinical rating Good/Fair		Clinical rating Poor				
		Mean	SD	Mean	SD	Mean	SD	Mean	SD	Clinical rating	Sex	Sex* Clinical rating
Dominant leg	Pelvic Drop	3.88	2.600	8.25	3.772	7.45	1.978	9.74	3.301	< 0.001*	0.001*	0.360
	Hip Adduction	79.59	6.000	75.37	6.634	76.35	5.349	70.09	6.754	< 0.001*	0.004*	0.965
	Knee Valgus	3.92	2.361	3.04	1.905	5.08	2.312	5.11	2.872	0.540	0.003*	0.760
Non-dominant leg	Pelvic Drop	4.24	0.644	6.39	0.604	5.86	0.779	9.46	0.592	< 0.001*	< 0.001*	0.066
	Hip Adduction	82.75	6.30	76.89	6.38	78.39	5.94	70.68	7.88	< 0.001*	< 0.001*	0.252
	Knee Valgus	3.10	2.16	3.69	2.20	4.64	3.44	5.54	3.16	0.563	0.001*	0.788

* Significant differences (P < 0.05)

### The explanatory joint angle measurements power of FSDT clinical rating

A significant prediction model was developed based on the three measured variables (pelvic drop, hip adduction, knee valgus) (e.g., 0.01 < p < 0.05; 0.312 < Nagelkerke R Square < 0.512).

The three parameters together can explain the performance clinical rating in 64.3% (females, dominant leg) to 76.6% (males, dominant leg) of the cases (Supplementary 3). A significant prediction based on one angle only was found for the pelvic tilt of the dominant leg in males only (p = = 0.003).

## Discussion

Performance and functional tests such as FSDT are essential in assessing the individual’s ability to perform daily and sport activities (e.g. stepping up or down stairs, running, jumping). These activities are commonly performed post-injury before the individual resumes full activities or sports [1].

Our main finding was that clinical rating of FSDT is correlated with joint angle measurements of the pelvis and hip joints showing that individuals rated as “poor” had higher pelvic drop and lower hip adduction angles compared with individuals rated as “good/fair”.

Similar to our study design, Perrott et al. (2021), examined the kinematics of athletes with good and poor lumbopelvic stability based on clinical rating of single leg squat (SLS) and dip test. During SLS participants rated as “poor” rotated their pelvis and side flexed their trunk toward the trail leg, while during dip test these participants had greater pelvic obliquity) [27].

Crossley et al. (2011), reported delayed onset of gluteus medius activity in individuals with poor FSDT performance. This might explain the findings of our study. In support of this explanation, previous studies suggested that hip abductor weakness might influence the performance of the individual during single leg tasks such as FSDT, single leg squat, and single leg mini squat [2, 9, 14, 28]. Diminished eccentric hip abductor muscle strength has been associated with greater hip adduction and contralateral pelvic drop during a single leg mini-squat [8]. In addition, the extent of anticipatory gluteus medius activity was significantly correlated with pelvic drop [29]. Thus, neuromuscular control deficit might be the link between poor performance and differences in pelvic and hip joint angles.

We suggest that the major differences between individuals who performed well/fairly or poorly were mainly in the pelvic and hip joints. The knee was not much involved and did not show a large difference between individuals. This is also in agreement with Perrott et al. (2021) who found significant differences in pelvic obliquity and hip adduction, but no differences in knee or ankle joint between athletes who performed poorly or well in the single leg squat test [27].

This study found no differences between dominant and non-dominant legs in the FSDT results and in joint angle measurements. This is in line with other studies examining symmetry during functional tests. Vaisman et al. (2017) examined symmetry of maximal muscular power using measurement of flight height in healthy young adults during single-leg squat jump, finding no differences between dominant and non-dominant legs. Other studies also found no differences between dominant and non-dominant leg regarding joint movement, range of motion or muscle strength [30–33].

Due to the development of modern technologies worldwide, there is an increasing tendency to develop new means to assess the individual’s quality of movement and a desire for a more objective tool than relying only on visual assessment by the examiner/therapist. These technologies include 3-D motion analysis systems (e.g. VICON) and wireless inertial sensors [34]. However, most medical clinics are not equipped with these technologies, as they are usually expensive to purchase, require software expertise and finally, are not applicable in the clinical setting. A clinical evaluation is still commonly used by clinicians, therapists and trainers in order to assess the individual’s quality of movement during different functional tasks. Thus, it is important to examine the accuracy of this visualized assessment.

In addition, we found differences between males and females suggesting higher pelvic drop, hip adduction and knee valgus in females compared with males. Nakagawa et al. (2012), too, examined differences between the sexes claiming that kinematics and neuromuscular activation during movement are different between males and females [8]. Their study likewise revealed that females have a greater amount of hip adduction compared with males, similar to our results. Yet, they did not find a difference between the sexes in the pelvic drop measurement, while we found a higher pelvic drop angle among females compared with males. Other studies found a similar tendency of higher angles for trunk, pelvis, hip and knee among females compared to males in single leg tests [15, 16, 18]. Thus, it is important to examine and compare between the sexes when assessing performance tests due to the difference between males and females in kinematics during gait and movements.

Our sample included healthy young individuals. For a physical task such as the FSDT, a healthy population with good neuromuscular ability and no risk of falling was preferred. Similar to our study, Perrott et al. (2021), examined single leg squat tests among athletes and categorized them by quality of performance (poor/good /neither good nor poor). Most of their population (39/62) were rated as having neither good nor poor performance. Healthy or active adults have a wide range of quality of movement, not all performing well or having the best results on functional tests. Functional tests are used to diagnose those who perform poorly so that exercise programs can be adjusted to improve their performance [27].

FSDT studies can be difficult to compare due to the large variety of test names available in the literature (e.g. forward step down test, single leg squat, single limb mini squat) [14, 18, 35]. In a recent meta-analysis, it was found that even when studies reported the same test name., e.g., SLS, the test protocol was different in 10 out of 12 studies [28]. In addition, there are several different ways in which performance is graded or scaled (e.g. 2, 3, 4 or more point scale and the joints that are being examined [28]. Our study used the 3 point scale (good, fair, poor) and 4 body segments (trunk, pelvic, hip, knee) and overall impression, and followed Crossley et al. (2011) and Herman et al. (2016) [13, 14].

Our study has several limitations. The participants were healthy adults; thus, the conclusions only relate to healthy individuals. In addition, we observed that only a small number of participants was rated with a “good” performance during FSDT. This limited our ability to perform statistical analysis and required us to subgroup good performance score with fair. Future studies should also examine hip muscle strength in symptomatic populations and examination with advanced technologies or gold standard (such as VICON).

## Conclusions

This study showed that the clinical rating of FSDT is correlated with joint angle measurements suggesting that this assessment can be utilized in clinical practice. Individuals with poor quality performance of FSDT showed higher pelvic drop and hip adduction movement. Further studies examining different populations with diverse disorders or pathologies are essential.

### Clinical implications

Clinical implications of this study suggest that the FSDT can be utilized in clinical practice enabling clinicians to visually identify faulty movements, in order to adjust exercise programs to improve performance or enhance rehabilitation.

### Electronic supplementary material

Below is the link to the electronic supplementary material.

Supplementary Material 1

Supplementary Material 2

Supplementary Material 3

## Data Availability

All data generated or analyzed during this study are included in this published article.

## Abbreviations

FSDT: Forward Step Down Test

## References

[1] Dar G, Yehiel A, Cale’ Benzoor M (2019). Concurrent criterion validity of a novel portable motion analysis system for assessing the landing error scoring system (LESS) test. Sports Biomech.

[2] Ageberg E, Bennell KL, Hunt MA, Simic M, Roos EM, Creaby MW (2010). Validity and inter-rater reliability of medio-lateral knee motion observed during a single-limb mini squat. BMC Musculoskelet Disord.

[3] Yamazaki J, Muneta T, Ju YJ, Sekiya I (2010). Differences in kinematics of single leg squatting between anterior cruciate ligament-injured patients and healthy controls. Knee Surg Sports Traumatol Arthrosc.

[4] Boling MC, Padua DA, Marshall SW, Guskiewicz K, Pyne S, Beutler A (2009). A prospective investigation of Biomechanical Risk factors for Patellofemoral Pain Syndrome: the Joint Undertaking to monitor and prevent ACL Injury (JUMP-ACL) cohort. Am J Sports Med.

[5] Powers CM (2010). The influence of abnormal hip mechanics on knee injury: a biomechanical perspective. J Orthop Sports Phys Ther.

[6] Alvim FC, Muniz AM, de Lucareli S, Menegaldo PRG (2019). Kinematics and muscle forces in women with patellofemoral pain during the propulsion phase of the single leg triple hop test. Gait Posture.

[7] Schmidt E, Harris-Hayes M, Salsich GB (2019). Dynamic knee valgus kinematics and their relationship to pain in women with patellofemoral pain compared to women with chronic hip joint pain. J Sport Health Sci.

[8] Nakagawa TH, Moriya ETU, Maciel CD, Serrão FV (2012). Trunk, pelvis, hip, and knee kinematics, hip strength, and gluteal muscle activation during a single-leg squat in males and females with and without patellofemoral pain syndrome. J Orthop Sports Phys Ther.

[9] Claiborne TL, Armstrong CW, Gandhi V, Pincivero DM (2006). Relationship between hip and knee strength and knee valgus during a single leg squat. J Appl Biomech.

[10] DiMattia MA, Livengood AL, Uhl TL, Mattacola CG, Malone TR (2005). What are the validity of the single-Leg-Squat Test and its relationship to hip-abduction strength?. J Sport Rehabilitation.

[11] Spinoso DH, Bellei NC, Marques NR, Navega MT (2018). Quadriceps muscle weakness influences the gait pattern in women with knee osteoarthritis. Adv Rheumatol.

[12] Park KM, Cynn HS, Choung SD (2013). Musculoskeletal predictors of movement quality for the forward step-down test in asymptomatic women. J Orthop Sports Phys Ther.

[13] Herman G, Nakdimon O, Levinger P, Springer S (2016). Agreement of an evaluation of the Forward-Step-Down Test by a broad cohort of clinicians with that of an Expert Panel. J Sport Rehabil.

[14] Crossley KM, Zhang WJ, Schache AG, Bryant A, Cowan SM (2011). Performance on the single-leg squat task indicates hip abductor muscle function. Am J Sports Med.

[15] Earl JE, Monteiro SK, Snyder KR (2007). Differences in Lower Extremity Kinematics between a bilateral Drop-Vertical Jump and a single-Leg step-down. J Orthop Sports Phys Therapy.

[16] Graci V, Van Dillen LR, Salsich GB (2012). Gender differences in trunk, pelvis and lower limb kinematics during a single leg squat. Gait Posture.

[17] Zeller BL, McCrory JL, Kibler WB, Uhl TL (2003). Differences in kinematics and electromyographic activity between men and women during the single-legged squat. Am J Sports Med.

[18] Weeks BK, Carty CP, Horan SA. Effect of sex and fatigue on single leg squat kinematics in healthy young adults. BMC Musculoskeletal Disorders [Internet]. 2015 Dec [cited 2020 Aug 2];16(1). Available from: http://bmcmusculoskeletdisord.biomedcentral.com/articles/10.1186/s12891-015-0739-3. 10.1186/s12891-015-0739-3 PMC4590784 26423154

[19] Dar G, Masharawi Y, Peleg S, Steinberg N, May H, Medlej B (2011). The Epiphyseal Ring: a long Forgotten Anatomical structure with significant physiological function. Spine.

[20] Kingston B, Murray A, Norte GE, Glaviano NR (2020). Validity and reliability of 2-dimensional trunk, hip, and knee frontal plane kinematics during single-leg squat, drop jump, and single-leg hop in females with patellofemoral pain. Phys Ther Sport.

[21] Whatman C, Hume P, Hing W (2013). The reliability and validity of physiotherapist visual rating of dynamic pelvis and knee alignment in young athletes. Phys Ther Sport.

[22] De Blaiser C, De Ridder R, Willems T, Danneels L, Roosen P (2019). Reliability of two functional clinical tests to evaluate trunk and lumbopelvic neuromuscular control and proprioception in a healthy population. Braz J Phys Ther.

[23] Willson JD, Davis IS (2008). Lower extremity mechanics of females with and without patellofemoral pain across activities with progressively greater task demands. Clin Biomech (Bristol Avon).

[24] Herrington L, Munro A (2010). Drop jump landing knee valgus angle; normative data in a physically active population. Phys Ther Sport.

[25] Landis JR, Koch GG (1977). An application of hierarchical kappa-type statistics in the assessment of majority agreement among multiple observers. Biometrics.

[26] Cicchetti DV (1994). Guidelines, criteria, and rules of thumb for evaluating normed and standardized assessment instruments in psychology. Psychol Assess.

[27] Perrott MA, Pizzari T, Opar MS, Cook J (2021). Athletes with a clinical rating of good and poor lumbopelvic stability have different kinematic variables during single leg squat and dip test. Physiother Theory Pract.

[28] Ressman J, Grooten WJA, Rasmussen Barr E (2019). Visual assessment of movement quality in the single leg squat test: a review and meta-analysis of inter-rater and intrarater reliability. BMJ Open Sport Exerc Med.

[29] Kim D, Unger J, Lanovaz JL, Oates AR (2016). The relationship of Anticipatory Gluteus Medius activity to pelvic and knee Stability in the transition to single-Leg stance. PM&R.

[30] Vaisman A, Guiloff R, Rojas J, Delgado I, Figueroa D, Calvo R (2017). Lower limb symmetry: comparison of muscular power between Dominant and nondominant legs in healthy young adults Associated with single-Leg-Dominant sports. Orthop J Sports Med.

[31] Ksoll KSH, Cotic M, Schmalzl K, Beitzel K, Achtnich A, Imhoff A (2022). Movement Coordination during functional single-Leg Squat tests in healthy, recreational athletes. Symmetry.

[32] DeLang MD, Kondratek M, DiPace LJ, Hew-Butler T, COLLEGIATE MALE SOCCER PLAYERS EXHIBIT BETWEEN-LIMB (2017). SYMMETRY IN BODY COMPOSITION, MUSCLE STRENGTH, AND RANGE OF MOTION. Int J Sports Phys Ther.

[33] McCurdy K, Langford G (2005). Comparison of unilateral squat strength between the dominant and non-dominant leg in men and women. J Sports Sci Med.

[34] Solarino G, Bortone I, Vicenti G, Bizzoca D, Coviello M, Maccagnano G (2021). Role of biomechanical assessment in rotator cuff tear repair: arthroscopic *vs* mini-open approach. WJO.

[35] Nae J, Creaby MW, Nilsson G, Crossley KM, Ageberg E (2017). Measurement Properties of a test Battery to assess Postural Orientation during Functional tasks in patients undergoing Anterior Cruciate Ligament Injury Rehabilitation. J Orthop Sports Phys Ther.

