# Supplementary figures and images for: Forward step down test - clinical rating is correlated with joint angles of the pelvis and hip: an observational study
Source: BMC Musculoskelet Disord. 2023 Oct 12;24:807. doi: 10.1186/s12891-023-06943-4 (PMC10568835; doi:10.1186/s12891-023-06943-4)

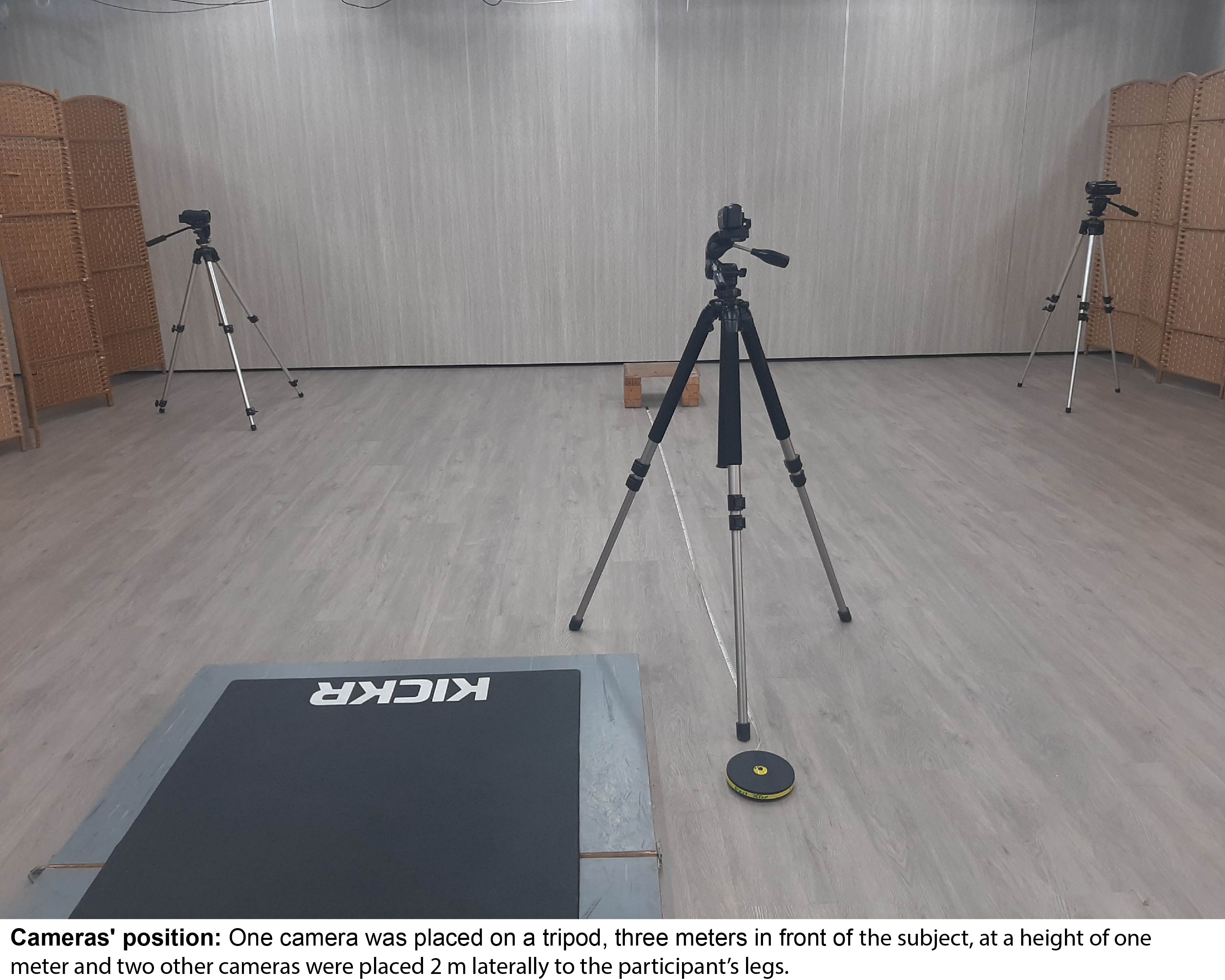

Supplement: Supplementary file 1 — Supplementary Material 1 [file 12891_2023_6943_MOESM1_ESM.jpg]
