# Supplementary material for: Forward step down test - clinical rating is correlated with joint angles of the pelvis and hip: an observational study
Source: BMC Musculoskelet Disord. 2023 Oct 12;24:807. doi: 10.1186/s12891-023-06943-4 (PMC10568835; doi:10.1186/s12891-023-06943-4)
Supplement: Supplementary file 3 — Supplementary Material 3 [file 12891_2023_6943_MOESM3_ESM.docx]

The binomial logistic regression analysis models.

|  | | Variable |  |  |  |  |  |  | 95% C.I.for EXP(B) | | ꭕ2 | R^2^ | %Correct |
| --- | --- | --- | --- | --- | --- | --- | --- | --- | --- | --- | --- | --- | --- |
|  |  |  | B | S.E. | Wald | df | Sig. | Exp(B) | Lower | Upper |  |  |  |
| Dominant leg | Males | Pelvic Drop | -0.562 | 0.190 | 8.765 | 1 | 0.003* | 0.570 | 0.393 | 0.827 | <0.001* | 0.501 | 76.6 |
|  |  | Hip Adduction | -0.008 | 0.065 | 0.014 | 1 | 0.905 | 0.992 | 0.874 | 1.126 |  |  |  |
|  |  | Knee Valgus | -0.229 | 0.182 | 1.581 | 1 | 0.209 | 0.795 | 0.556 | 1.137 |  |  |  |
|  | Females | Pelvic Drop | -0.205 | 0.170 | 1.463 | 1 | 0.226 | 0.814 | 0.584 | 1.136 | 0.011* | 0.312 | 64.3 |
|  |  | Hip Adduction | -0.133 | 0.070 | 3.590 | 1 | 0.058 | 0.875 | 0.763 | 1.005 |  |  |  |
|  |  | Knee Valgus | -0.019 | 0.155 | 0.015 | 1 | 0.901 | 0.981 | 0.724 | 1.328 |  |  |  |
| Non-Dominant leg | Males | Pelvic Drop | -0.201 | 0.126 | 2.534 | 1 | 0.111 | 0.818 | 0.639 | 1.047 | 0.004* | 0.335 | 74.5 |
|  |  | Hip Adduction | -0.107 | 0.063 | 2.875 | 1 | 0.090 | 0.899 | 0.794 | 1.017 |  |  |  |
|  |  | KneeValgus | 0.210 | 0.177 | 1.406 | 1 | 0.236 | 1.233 | 0.872 | 1.744 |  |  |  |
|  | Females | Pelvic Drop | -0.235 | 0.154 | 2.323 | 1 | 0.127 | 0.791 | 0.585 | 1.069 | 0.006* | 0.358 | 73.2 |
|  |  | Hip Adduction | -0.083 | 0.065 | 1.609 | 1 | 0.205 | 0.920 | 0.809 | 1.046 |  |  |  |
|  |  | Knee Valgus | 0.027 | 0.122 | 0.050 | 1 | 0.824 | 1.028 | 0.808 | 1.306 |  |  |  |

*Significant difference: ꭕ2<0.05 for a prediction based on 3 angular measurements, p<0.05 for a prediction based on one angular measurement.
